# Supplementary material for: Evidence supported by Mendelian randomization: impact on inflammatory factors in knee osteoarthritis
Source: Front Med (Lausanne). 2024 May 28;11:1382836. doi: 10.3389/fmed.2024.1382836 (PMC11165061; doi:10.3389/fmed.2024.1382836)
Supplement: Supplementary file 1 [file Data_Sheet_1.ZIP › data/GCST90274791_5e6/791.leaveoneout.pdf]

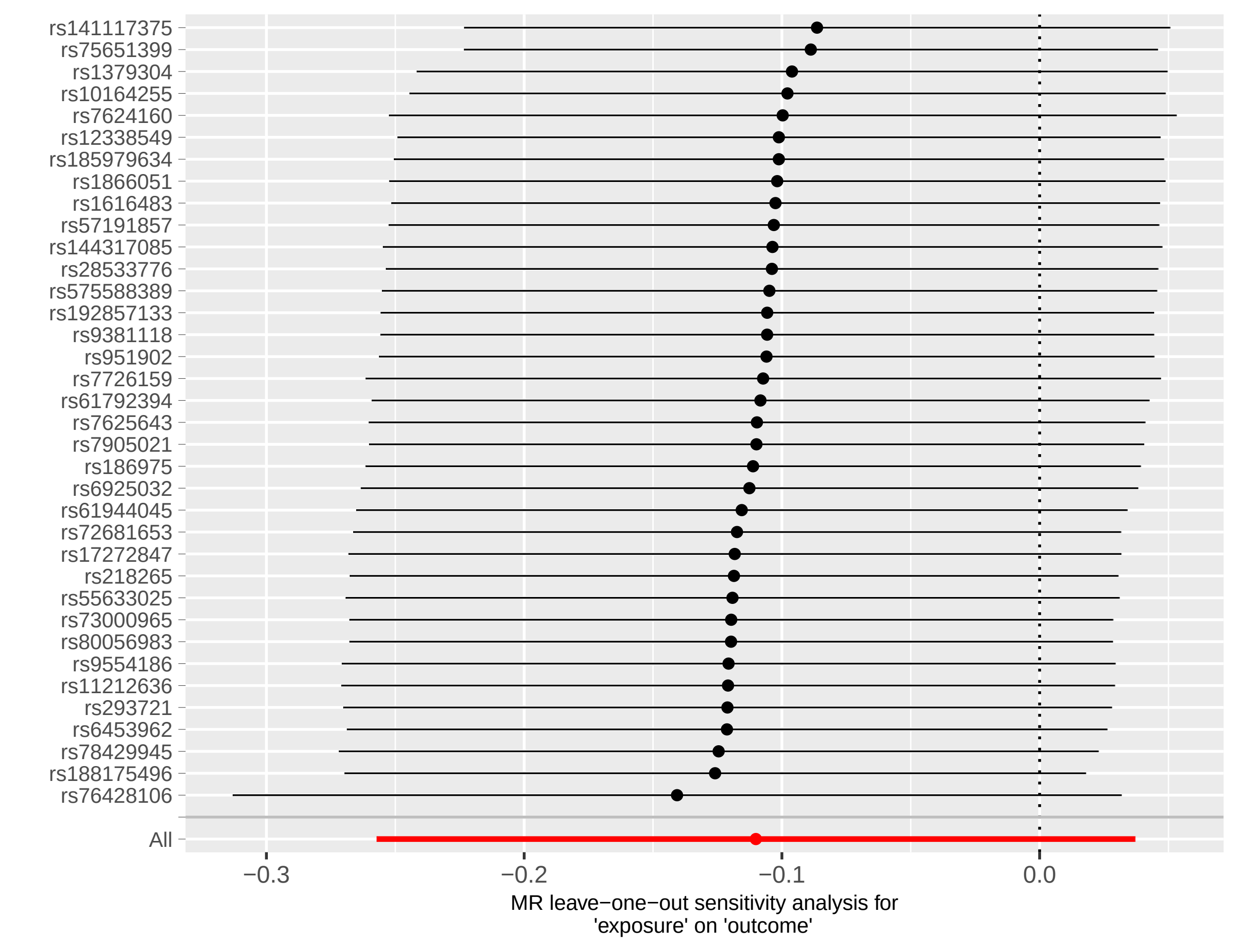
rs141117375
rs75651399
rs1379304
rs10164255
rs7624160
rs12338549
rs185979634
rs1866051
rs1616483
rs57191857
rs144317085
rs28533776
rs575588389
rs192857133
rs9381118
rs951902
rs7726159
rs61792394
rs7625643
rs7905021
rs186975
rs6925032
rs61944045
rs72681653
rs17272847
rs218265
rs55633025
rs73000965
rs80056983
rs9554186
rs11212636
rs293721
rs6453962
rs78429945
rs188175496
rs76428106
All
−0.3
−0.2
−0.1
0.0
MR leave−one−out sensitivity analysis for
'exposure' on 'outcome'
